# Development of an Amperometric-Based Glucose Biosensor to Measure the Glucose Content of Fruit

**DOI:** 10.1371/journal.pone.0111859

**Published:** 2015-03-19

**Authors:** Lee Fung Ang, Lip Yee Por, Mun Fei Yam

**Affiliations:** 1 School of Pharmaceutical Sciences, Universiti Sains Malaysia, 11800, Penang, Malaysia; 2 Faculty of Computer Science and Information Technology, University of Malaya, 50603, Kuala Lumpur, Malaysia; Leibniz-Institute for Vegetable and Ornamental Crops, GERMANY

## Abstract

An amperometric enzyme-electrode was introduced where glucose oxidase (GOD) was immobilized on chitosan membrane via crosslinking, and then fastened on a platinum working electrode. The immobilized enzyme showed relatively high retention activity. The activity of the immobilized enzyme was influenced by its loading, being suppressed when more than 0.6 mg enzyme was used in the immobilization. The biosensor showing the highest response to glucose utilized 0.21 ml/cm^2^ thick chitosan membrane. The optimum experimental conditions for the biosensors in analysing glucose dissolved in 0.1 M phosphate buffer (pH 6.0) were found to be 35°C and 0.6 V applied potential. The introduced biosensor reached a steady-state current at 60 s. The apparent Michaelis-Menten constant ($K_{M}^{app}$) of the biosensor was 14.2350 mM, and its detection limit was 0.05 mM at s/n > 3, determined experimentally. The RSD of repeatability and reproducibility of the biosensor were 2.30% and 3.70%, respectively. The biosensor was showed good stability; it retained ~36% of initial activity after two months of investigation. The performance of the biosensors was evaluated by determining the glucose content in fruit homogenates. Their accuracy was compared to that of a commercial glucose assay kit. There was no significance different between two methods, indicating the introduced biosensor is reliable.

## Introduction

Biosensors have wide applications ranging from the food industry to environmental monitoring and clinical analysis. The concept of a biosensor was first introduced by Clark and Lyons [1] in the form of an oxygen electrode for monitoring glucose. An electrochemical biosensor has been defined as “a self-contained integrated device, which is capable of providing specific quantitative and semi-quantitative analytical information using a biological recognition element (biochemical receptor) which is retained in direct spatial contact with an electrochemical transduction element” [2]. It should respond to analytes selectively, continuously, rapidly, specifically and ideally without any added reagent. Enzymes, antibodies, nucleic acids and receptors are the four main groups of biological elements encountered in biosensors, with enzymes being the most regularly employed.

About half of the published papers on biosensors are related to glucose monitoring, primarily due to its metabolic and medical importance [3–7]. It also serves as a good analyte for the development of new biosensors. In the Clark oxygen electrode, glucose oxidase (GOD) was retained by a perm-selective membrane adjacent to an amperometric detector as the sensing element. GOD, a highly specific enzyme, is the most widely studied of all amperometric-based enzymes for biosensors. This enzyme catalyses the oxidation of glucose to gluconolactone according to the following reaction:

$$\beta \text{-D-glucose + GOD}\left(\text{FAD}\right)⇌\text{GOD}\left({\text{FADH}}_{\text{2}}\right)\text{ + D-glucono-δ-lactone}$$ (reaction 1)

GOD catalyzes the oxidation of β-D-glucose to D-glucono-δ-lactone and hydrogen peroxide (H_2_O_2_) using molecular oxygen (O_2_) as the electron acceptor. The two-stage enzyme process, typical for the class of oxidases, consists of enzymatic oxidation of glucose by its cofactor FAD (flavin adenine dinucleotide) (redox centre) which is then reduced to FADH_2_ (reaction 1).

This is followed by its reoxidation or regeneration of the biocatalyst by O_2_ with formation of H_2_O_2_ (reaction 2):

$$\text{GOD}\left({\text{FADH}}_{\text{2}}\right){\text{ + O}}_{\text{2}}\to \text{ GOD}\left(\text{FAD}\right){\text{ + H}}_{\text{2}}{\text{O}}_{\text{2}}$$ (reaction 2)

The D-glucono-δ-lactone produced in the reaction (1.1) is a weak competitive inhibitor of glucose, which hydrolyses spontaneously to gluconic acid (reaction 3).

$${\text{D-glucono-δ-lactone+H}}_{\text{2}}\text{O}\;\to \;\text{gluconic}\;\text{acid}$$ (reaction 3)

The overall reaction is expressed as:

$$\beta \text{-D-glucose}+{\text{O}}_{2}+{\text{H}}_{2}\text{O}\overset{\text{GOD}}{\to }\text{gluconic}{\text{acid}}_{}+{\text{H}}_{2}{\text{O}}_{2}$$ (reaction 4)

Although specific for β-D-glucose, GOD can be used to measure total glucose because α-glucose is converted to the β-form by mutarotation at equilibrium. Thus, GOD is widely used for the determination of free glucose in body fluids.

In amperometry, the detection of glucose is usually based on measuring the increase in the anodic current (H_2_O_2_ oxidation) or the decrease in the cathodic current (O_2_ reduction) at the electrochemical cell at a fixed potential. Oxygen electrode-based glucose biosensors and hydrogen peroxide electrode-based glucose biosensors are two commonly studied amperometric glucose biosensors. The reactions on a cathodically polarized platinum electrode are shown below:

$${\text{O}}_{\text{2}}{\text{+ 2H}}^{\text{+}}{\text{+ 2e}}^{\text{-}}\overset{\text{Pt}}{\to }{\text{ H}}_{\text{2}}{\text{O}}_{\text{2}}$$ (reaction 5)

$${\text{H}}_{\text{2}}{\text{O}}_{\text{2}}{\text{+ 2H}}^{\text{+}}{\text{+ 2e}}^{\text{-}}\overset{\text{Pt}}{\to }{\text{2H}}_{\text{2}}\text{O}$$ (reaction 6)

The basic principle of the first-generation of glucose biosensors is the quantification of glucose through electrochemical detection of the enzymatically liberated H_2_O_2_, where the current produced is proportional to its concentration. In an amperometric glucose biosensor, the working potential over which H_2_O_2_ is detected is typically between 500–750 mV vs. Ag/AgCl.

There are different procedures for immobilizing the biological component in a thin layer at the transduction surface of electrochemical biosensors. For instance, Low et al. [8] demonstrated the preparation of ferrocene-containing photopolymeric films based on hydrophilic methacrylate polymer, which can prevent leaching of both ferrocene and enzyme. A new glucose biosensor based on covalent immobilization of GOD on carbon nanotube film functionalized with carboxylic acid groups was described by Xue et al. [9]. Subsequently, Lim et al. [10] reported a glucose biosensor based on electrochemical co-deposition of palladium nanoparticles and GOD onto a carbon nanotube film. A novel glucose biosensor utilizing nanoporous ZrO_2_/chitosan composite film as an immobilization matrix for GOD was developed by Yang et al. [11]. Another technique involving Langmuir-Blodgett film deposition of a conducting organic polymer poly (3-dodecyl thiophene) to immobilize GOD for glucose biosensing was reported by Singhal et al. [12].

The properties of immobilized enzymes are governed by the properties of both the enzyme and the support material. Generally, the support material should possess some of the desirable characteristics such as high affinity to proteins, ease of chemical modifications, hydrophilicity, mechanical stability and rigidity, possibility of regeneration so as to provide the system with a permeable surface suitable for a chosen biotransformation [13]. In recent years, chitosan has been widely used as a support for enzyme immobilization in the construction of biosensors [14–20].

Chitosan is poly[-(1–4)-2-amino-2-deoxy-ß-D-glucopyranose], a cationic polysaccharide abundant in the shells of crustaceans. It is derived by partial deacetylation of chitin [21]. The presence of amino groups gives it a basic character. It is an ideal immobilization matrix for the fabrication and construction of biosensors, with properties including excellent membrane-forming ability, high water permeability, good adhesion, biocompatibility, biodegradability, antibacterial properties, lack of toxicity, heavy metal ion chelation, hydrophilicity and a remarkable affinity for proteins due to the presence of reactive amino and hydroxyl functional groups [13,20,22]. Its ability to absorb metal ions and various organic halogen substances can also prevent the immobilized enzyme used in biosensors from damage [23]. In addition, chitosan can form a thermally and chemically inert film that is insoluble in water [23].

The sugar (fructose, sucrose and glucose) content in ripened fruits is correlated with their glycaemic index. Diabetic patients often question and worry whether it is safe for them to eat fruit, which can contain large quantities of sugar. Glucose is a major monosaccharide found in almost all fruits and is easily absorbed through gastrointestinal tract to increase blood glucose levels. Thus, the glucose content attracts great attention as an indicator of the glycaemic index for fruits. Therefore, the development of simple, reliable and economical glucose biosensors is desirable for measuring the glucose content of fruits. Application of such biosensor for quality control in the fruit industry can have an economic impact as well as health benefits to end users who are diabetic patients. In this paper, we report the fabrication and characterization of an amperometric-based glucose biosensor for measuring glucose content in fruit homogenates.

## Materials and Methods

### 2.1 Materials

Glucose oxidase from *Aspergillus niger* (EC 1.1.3.4, type VII, 185,000 units/g solid), glutaraldehyde (grade II, 25% aqueous solution) and glycerol were purchased from Sigma (St. Louis, USA). Chitosan (Code #22742) was procured from Fluka (Switzerland). D(+)glucose monohydrate was supplied by System (Malaysia). Glacial acetic acid, citric acid monohydrate and potassium phosphate (KH_2_PO_4_) were obtained from R&M Marketing (Essex, U.K) while di-sodium hydrogen phosphate dehydrate (Na_2_HPO_4_·2H_2_O) and sodium chloride were bought from Hamburg Chemical (Germany). Tri-sodium citrate was purchased from Grauwmeer (Leuven, Belgium). Hydrogen peroxide (>30% w/v) was bought from Fisher Scientific (Loughborough, UK), while premounted dialysis membrane (Ezee-Mount, type “C”) was bought from Fisher Scientific (Pittsburgh, Pennsylvania, USA). Aluminium oxide (highly pure for polishing) was purchased from BDH Laboratory Supplies (England). A PK-4 Polishing kit (MF-2060), platinum working electrode (MF-2013), and silver/silver chloride (Ag/AgCl) reference electrode (MF-2079) were purchased from Bioanalytical Systems Inc. (West Lafayette, Indiana, USA). All chemicals were of analytical grade and were used without further purification. The molecular weight of chitosan was determined by dilute solution viscosity using an Ubbelohde viscometer U-tube (size C, VS-220, Technico, England) and the degree of deacetylation was determined using the first derivative UV-spectrophotometric method. The chitosan had viscosity-average molecular weight of 981.80 kDa and was 82.44% deacetylated.

### 2.2 Methods

**2.2.1 Electrochemical measurement.** Amperometric detection of glucose was performed using a potentiostat (CV-1B cyclic voltammograph, Bioanalytical Systems Inc. (BAS), West Lafayette, Indiana, USA) poised at +0.6 V connected to an integrator-plotter (D-2500 Chromato-Integrator, Hitachi, Tokyo, Japan) and a digital multimeter (8022A, Fluke, USA). The conventional three electrodes consisted of a silver/silver chloride (Ag/AgCl) reference electrode (MF-2079, BAS, West Lafayette, Indiana, USA), a platinum wire (0.25 mm diameter, 99.99%, Aldrich, Milwaukee, Wisconsin, USA) as counter electrode and a platinum working electrode (MF-2013, BAS, West Lafayette, Indiana, USA) with the enzyme-chitosan layer and a protective dialysis membrane. Unless stated otherwise, all experiments were carried out in 10 ml of phosphate buffer (0.1 M, pH 7.0) maintained at 25 ± 0.1°C using a digital temperature controller (Model 9001, Poly Science, USA) with stirring to provide convective transport. Glucose stock solution (prepared in phosphate buffer) was allowed to mutarotate at 4°C for at least 24 h prior to use, since only β-D-glucose is a substrate for the enzymatic reaction. At stable background current, aliquots of the β-D-glucose stock solution were introduced into the stirred phosphate buffer and the steady anodic current produced by the enzymatically generated H_2_O_2_ was recorded.

**2.2.2 Preparation of chitosan membrane and characterization.** One gram of chitosan was dissolved in 100 ml of 0.8% (w/v) acetic acid and stirred overnight to ensure complete dissolution. Varying volumes of chitosan solution were then pipetted into petri dishes at a pre-measured volume per surface area (ml/cm^2^) and then allowed to dry overnight in an oven at 60°C. The thickness of the membranes was measured using a micrometer (digimatic micrometer, Mitutoyo, Tokyo, Japan) at five locations (the centre and four corners), and the mean thickness calculated. Mechanical properties such as tensile strength and elongation at break were measured with a texture analyser (TA.XT2, Stable Micro System, Haslemere, Surrey, UK) equipped with a 5 kg load cell. The other prepared membranes were neutralized with 1% w/v sodium hydroxide (NaOH) for 30 minutes followed by rinsing with distilled water to remove excess NaOH. The neutralized membranes were cut into squares (1.5 x 1.5 cm^2^) for the diffusion study and for enzyme immobilization.

**2.3.3 Study on the diffusion of hydrogen peroxide through chitosan membranes.** The diffusion properties of chitosan membranes cast in different thicknesses were determined by measuring the electrode response to H_2_O_2_ using amperometric detection. The anodic current generated by H_2_O_2_ which had diffused through the membrane was sensed by (i) a bare platinum electrode (bare PT), (ii) an electrode covered with dialysis membrane (Dialysis PT) and (iii) an electrode covered with blank chitosan membrane in addition to a dialysis membrane (CHIT/PT). A comparison of the effect of the chitosan membranes on the electrode response to H_2_O_2_ was then made.

**2.2.4 Enzyme immobilization.** The method of Magalhães et al. [24] was modified to immobilize the GOD onto the chitosan membrane. One side of the square of membrane (with a thickness of 0.35 ml/cm^2^) was coated with 20 μl of 1% (v/v) glutaraldehyde and allowed to dry at room temperature. Subsequently, 20 μl of 10 mg/ml (0.2 mg) GOD in phosphate buffer (0.1 M, pH 7.0) containing 5% (v/v) glycerol was spread evenly onto the same surface of the membrane with the aid of an L-shaped rod. The immobilized membrane was then left to dry at room temperature. The small amount of glycerol in the enzyme solution acts as an emollient to facilitate even spreading of GOD on the membrane surface. The dried membrane was washed with distilled water and kept in phosphate buffer (0.1 M, pH 7.0) at 4°C until further use.

**2.2.5 Construction of the glucose biosensor.** The platinum electrode was first polished with 0.05 μm alumina on a polishing pad, washed with distilled water and finally sonicated for 2 minutes to remove the alumina particles. Then the GOD-chitosan membrane and a moist dialysis membrane as a lamination layer were fastened onto the surface of the platinum electrode with an O-ring.

**2.2.6 Optimization of experimental variables for the analysis of glucose using biosensor.** The factors influencing enzymatic activity and ultimately biosensor performance were investigated. These included applied potential, membrane thickness, glutaraldehyde concentration, enzyme concentration, temperature, pH and buffer concentration.

The effect of applied potential on the steady-state current response of the enzyme-electrode in the potential range from 0.30–0.80 V in 0.05 V increments was studied. The potential was set at the lowest voltage of 0.30 V and the background current allowed to decay to a steady-state value before increasing the applied potential stepwise to 0.80 V. A comparison of the response current at different applied potentials generated by the bare platinum electrode in phosphate buffer and in 0.05 mM H_2_O_2_ in phosphate buffer as well as by the biosensor (GOD-CHIT/PT) in 2 mM glucose in phosphate buffer was made.

The fabricated chitosan membranes were cast in thicknesses ranging from 0.21 to 0.42 ml/cm^2^. Glutaraldehyde concentration was investigated from 0.1% v/v to 1.0% v/v. Various amounts of GOD (0.05–0.8 mg) in phosphate buffer (0.1 M, pH 7.0) were immobilised on the chitosan membrane. The effect of the temperature of analysis (from 15–50°C) on biosensor performance was studied by measuring the anodic current generated. The optimal pH for enzymatic activity was investigated by varying the pH value from 4.0–8.0. Buffers at different pH values were prepared with 0.1 M citrate buffer to obtain pH values from 4.0–5.5 and with 0.1 M phosphate buffer to attain pH values in the 6.0–8.0 range. The concentration of the working buffer at optimum pH was investigated from 0.01–0.20 M. The optimal value obtained for each parameter was used in subsequent experiments.

**2.2.7 Calibration of the glucose biosensor.** Six GOD-CHIT/PT electrodes were prepared according to the optimal conditions: 0.6 mg GOD was immobilized onto 0.21 ml/cm^2^ chitosan membrane and crosslinked with 0.2% v/v glutaraldehyde. The biosensors performed their electrochemical measurements at 35°C, in a supporting electrolyte of 0.1 M phosphate buffer, pH 6.0.

Aliquots of β-D-glucose stock solution were successively added into 10 ml of stirred phosphate buffer in an electrochemical cell. Three different glucose stock solutions with concentrations of 0.01 M, 0.1 M and 1.0 M were prepared to obtain the hydrodynamic response for glucose from 0.01 to 130 mM. The mean value of the anodic current (μA) was plotted against the glucose concentration (in mM). The linear range of the biosensor was then determined from the saturation curve. The detection limit at which the signal to noise (s/n) > 3 was determined experimentally. The apparent Michaelis-Menten constant, $K_{M}^{app}$ and *I*_max_ were determined by from a Eadie-Hofstee plot using the equation shown below:

$$I=I_{\max}-K_{M}^{app}\left(\frac{I}{c}\right)$$

where *I* is the steady state current after the addition of substrate, *c* is the bulk concentration of the substrate (glucose) and *I*_max_ is the maximum current measured under a saturated substrate condition. The $K_{M}^{app}$ and *I*_max_ of the biosensor were then determined from the slope and intercept on the y-axis of the plot.

**2.2.8 Repeatability and reproducibility.** The repeatability generated by the glucose biosensor was studied by measuring the anodic current generated by 3.98 mM glucose in 10 ml phosphate buffer (pH 6.0) a total of 20 times in a single day. On the other hand, the reproducibility of the biosensors was studied by measuring the current generated by 3.98 mM glucose in 10 ml phosphate buffer (pH 6.0) by using six different glucose biosensors. Each biosensor was tested by replicate (n = 3) analysis. The total mean value was calculated and the relative standard deviation (RSD) provided the analytical precision. The RSD was calculated using the following equation:

$$\text{RSD(\%)= }\frac{\text{Standard deviation}}{\text{Average}}\times 100\%$$

**2.2.9 Storage stability study.** Three good GOD-CHIT/PT biosensors were prepared. On the other hand, three free enzyme-electrodes (GOD/PT) were prepared with the same amount of GOD, where the enzyme was coated directly on the platinum electrode surface without immobilization, only protected with a layer of dialysis membrane and fastened with an O-ring. The storage stability of the two types of enzyme-electrodes was explored under optimal experimental conditions.

The responses of the three GOD-CHIT/PTs and three GOD/PTs to 3.98 mM glucose were measured daily during the first two weeks. After 2 weeks, the biosensors were tested every 3–5 days over a period of 80 days. The mean of the relative current to the initial current sensed by these biosensors was plotted as a function of time. All enzyme electrodes were kept in 0.1 M phosphate buffer (pH 7.0) and stored at 4°C when not in use.

**2.2.10 Glucose determination in fruit.** Fresh fruits (banana, watermelon, orange, mango, apple and pear) were obtained from the local market and homogenized with distilled water (10 or 1 g in 1 L). Further dilution using distilled water was made if necessary. The homogenate was centrifuged at 3000 rpm and 4°C and the supernatant was filtered through a nylon syringe filter (0.2 μm) prior to determining the glucose content using the glucose biosensor and a commercial glucose assay kit (Sigma-Aldrich, USA) that relies on spectrophotometric detection. The statistical analysis method (T-test) was used to compare the results obtained from the glucose biosensor and the commercial glucose assay kit at a level of significance *P<*0.05 using GraphPad v5.

## Results and Discussion

### 3.1 Characterization of chitosan membrane

**3.1.1 Mechanical properties.** A concentration of acetic acid higher than 0.8% w/v was not suitable for use in enzyme immobilization [23]. Thus, 1 g of chitosan was dissolved in 100 ml of 0.8% w/v acetic acid solution. Chitosan membranes were prepared by casting the solution in a petri dish in different measured volumes, i.e. from 0.21–0.42 ml/cm^2^. The mechanical strength of the membranes was described in terms of tensile strength whilst their brittleness was characterized by a decrease in the percentage of elongation at break. As shown in Table 1, the thicker the membrane the greater the force required to break it. However, the values of elongation at break decreased with increasing the membrane thickness, implying a reverse correlation between the brittleness and thickness of the membranes.

**Table 1:** Mechanical property of chitosan membranes with different thicknesses.

Casting measurements (ml/cm^2^)	Membrane thickness (mm)^a^	Tensile Strength (N/mm^2^) ^a^	Elongation at Break (%/mm^2^) ^a^
0.21	0.013 ± 0.003	85.907 ± 7.346	35.569 ± 6.424
0.28	0.020 ± 0.001	90.228 ± 6.143	22.174 ± 4.124
0.35	0.021 ± 0.001	91.439 ± 5.932	21.062 ± 1.959
0.42	0.028 ± 0.003	92.988 ± 7.031	20.226 ± 5.975

^a^Mean ± SD, n = 6

**3.1.2 Studies on the diffusion of hydrogen peroxide through chitosan membranes.** The effect of different membrane thicknesses on diffusion of the substrate was studied using H_2_O_2_ as an analyte at pH 7.0. The electrodes used were a bare platinum electrode (bare PT), a platinum electrode covered with dialysis membrane (Dialysis/PT) and a third platinum electrode covered with both chitosan and dialysis membrane (CHIT/PT). The diffusion barrier was calculated as a permeation factor and expressed as a percentage as shown in the following equation:

$$\text{Membrane}\;\text{permeation}\;\text{factor}\;(\%)\;=\;\frac{{\text{Response}\;\text{of}\;\text{CHIT}/\text{PT}\;\text{or}\;\text{Dialysis/PT}\;\text{to}\;\text{H}}_{2}{\text{O}}_{\text{2}}}{{\text{Response}\;\text{of}\;\text{bare}\;\text{PT}\;\text{to}\;\text{H}}_{2}{\text{O}}_{\text{2}}}\times 100\%$$

Table 2 shows that the main diffusion barrier was due to the presence of the dialysis membrane (~31.56%) used as a protective layer on the surface of the working electrode. With increasing thickness of the chitosan membrane, the response and permeation factor were accordingly decreased. Although thinner membranes were associated with a diminished diffusion barrier, the membrane with a measured volume of 0.17 ml/cm^2^ was too thin. It was not only brittle but also difficult to handle.

**Table 2:** Effect of membrane thickness on electrode response to 0.5 mM H_2_O_2_.

Electrode type	^a^Bare PT	aDialysis/PTmembrane	^b^CHIT/PT 1	^b^CHIT/PT 2	^b^CHIT/PT 3	^b^CHIT/PT 4	^b^CHIT/PT 5	^b^CHIT/PT6	^b^CHIT/PT 7
**Casting measurements (ml/cm** ^**2**^ **)**			0.17	0.21	0.24	0.28	0.31	0.35	0.42
**Current (μA)**	0.995 ± 0.120	0.681 ± 0.087	0.487 ± 0.014	0.476 ± 0.005	0.476 ± 0.003	0.345 ± 0.009	0.321 ± 0.020	0.310 ± 0.023	0.284 ±0.007
**Permeation factor (%)**	100	68.44	48.94	47.84	47.84	34.67	32.26	31.16	28.54
^**#**^ **LSD (Statistical significance)**	*P*<0.05: CHIT4/PT & CHIT5/PT, /CHIT6/PT & CHIT7PT; *P*<0.01: CHIT4/ PT & CHIT6/PT; *P*<0.001: CHIT1/PT & CHIT4/PT, CHIT1/PT & CHIT5/PT, CHIT1/PT & CHIT6/PT, CHIT1/PT & CHIT7/PT, CHIT2/PT & CHIT4/PT, CHIT2/PT & CHIT5/PT, CHIT2/PT & CHIT6/PT, CHIT2/PT & CHIT7/PT, CHIT3/PT & CHIT4/Pt, CHIT3/PT & CHIT5/PT, CHIT3/PT & CHIT6/PT, CHIT3/PT & CHIT7/PT, CHIT4/PT & CHIT7/PT								

^a^Mean ± SD, n = 6;

^b^mean ± SD, n = 3.

^#^ Comparison of current among the CHIT-PT electrodes.

### 3.2 Optimization of experimental variables for glucose biosensor

**Fig 1:**
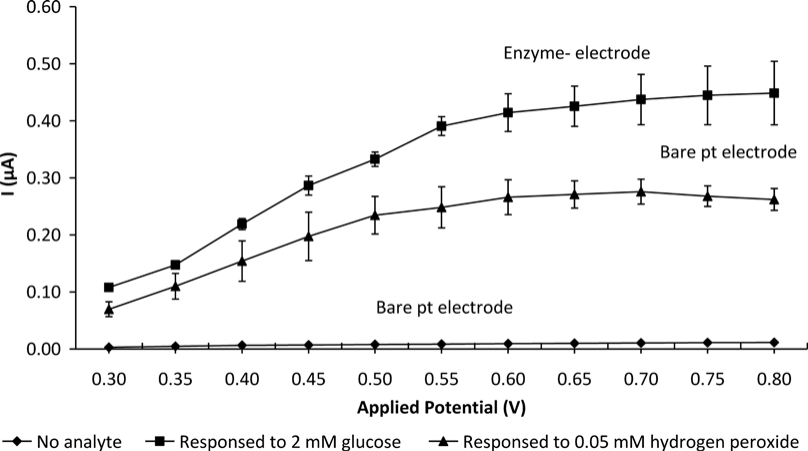
Effect of applied potential on the steady-state response with a bare platinum electrode in sensing 0.05 mM H_2_O_2_ and GOD-CHIT/PT in detecting 2 mM glucose. Phosphate buffer was used as the medium in both cases. Mean ± SD, n = 4.

**3.2.1 Selection of applied potential.** Fig. 1 compares the response current as a function of applied potential from 0.3 to 0.8 V obtained with a bare platinum electrode sensing 0.05 mM H_2_O_2_ and GOD-CHIT/PT detecting 2 mM glucose. Phosphate buffer was used as the medium in both cases. The anodic current curve of the enzyme electrode in sensing glucose demonstrated a behaviour similar to that of the bare platinum electrode in sensing H_2_O_2_ over the same applied potential. The response current for both sensors increased from 0.3 to 0.6 V and then plateaued. The background current of the electrode in phosphate buffer increased only slightly with increasing applied potential from 0.3 to 0.8 V. An applied potential of 0.6 V was chosen as the working potential in subsequent experiments as it gave the highest response for the electrodes.

**Fig 2:**
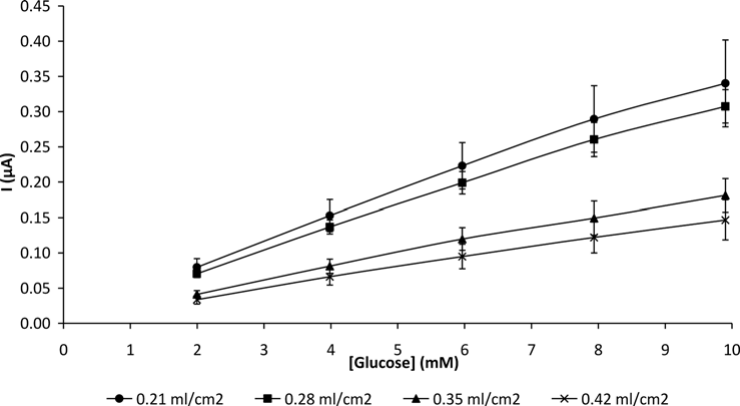
Effect of chitosan membrane thickness on GOD-CHIT/PT response for glucose in 0.1 M phosphate buffer (pH 7.0). Mean ± SD, n = 6.

**3.2.2 Effect of membrane thickness on biosensor response.** Chitosan membranes were cast in thicknesses ranging from 0.21 to 0.42 ml/cm^2^ and cut into 1.5 x 1.5 cm^2^ squares. The prepared membranes were immobilized with 10 mg/ml GOD and applied to the platinum electrode using the methods as stated in 2.2.4 and 2.2.5. The GOD-CHIT/PT electrodes were then utilized for electrochemical measurement. In general, the behaviour of an enzyme-electrode can be influenced by membrane thickness. The enzyme immobilized on a chitosan membrane with a measured volume of 0.21 ml/cm^2^ showed the highest response to glucose in amperometric detection. A decrease in current was observed with thicker membranes (Fig. 2).

The membrane prepared with a measured volume of 0.21 ml/cm^2^ was found to be ideal for enzyme immobilization because it was mechanically stable (Table 1) and could retain sufficient enzyme in its pores. Although very thin membranes presented smaller diffusion barriers, only insignificant amounts of enzyme might be immobilized. In addition, thin membranes have poor mechanical stability. However, a thick membrane might decrease mass transport of the substrate through the host matrix due to the higher diffusion barrier [23]. When the membrane is thick, diffusive hindrance to the substrate could result in a low and slow response (Table 2). The response of the enzyme electrodes was not only affected by the quantity of the enzyme immobilized but the permeability of the membrane as well.

In this study a dialysis membrane placed on top of the chitosan-enzyme membrane served as a protective barrier against the surrounding environment, thus preventing the chitosan membrane from breaking.

**Fig 3:**
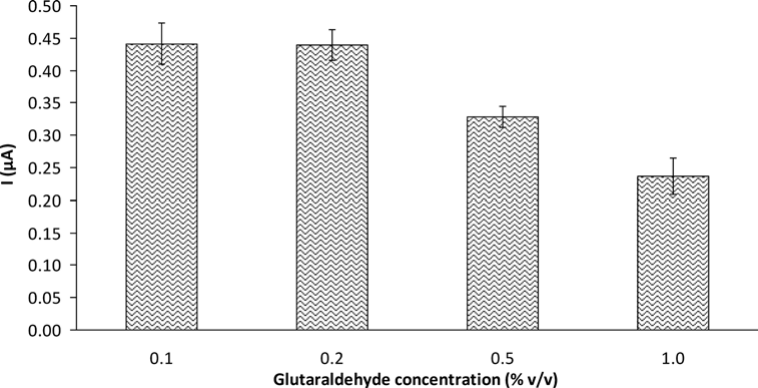
Effect of glutaraldehyde concentration on GOD-CHIT/PT response to 4.76 mM glucose (mean ± SD, n = 3).

**3.2.3 Effect of the glutaraldehyde concentration used for immobilization on biosensor response.** Glucose oxidase was immobilized onto chitosan membranes via glutaraldehyde crosslinking. The purpose of immobilization was to stabilize the tertiary structure of the protein and thus render it less sensitive to the external environment. The effect of the glutaraldehyde on the activity of the immobilized enzyme in the glucose biosensor is shown in Fig. 3. The current increased slightly as the glutaraldehyde concentration increased from 0.1% v/v to 0.2% v/v. Further increase in the glutaraldehyde concentration caused a decline in the current, indicating deactivation of the enzyme. Although the bifunctional—CHO groups of glutaraldehyde simultaneously react with—NH_2_ sites on the chitosan to facilitate bonding of the enzyme to chitosan, partial inactivation of the enzyme by glutaraldehyde can occur if the crosslinking agent is present in excess. Wang et al. [23] reported similar findings on deactivation of the enzyme by a high concentration of glutaraldehyde. Furthermore, at high concentration, the glutaraldehyde-treated membranes became deep yellow. The brittleness of the membrane increased with increasing glutaraldehyde concentration. This result was in agreement with that observed by Yang et al. [17]. Based on our findings, 0.2% v/v glutaraldehyde was used for subsequent enzyme immobilization.

**Fig 4:**
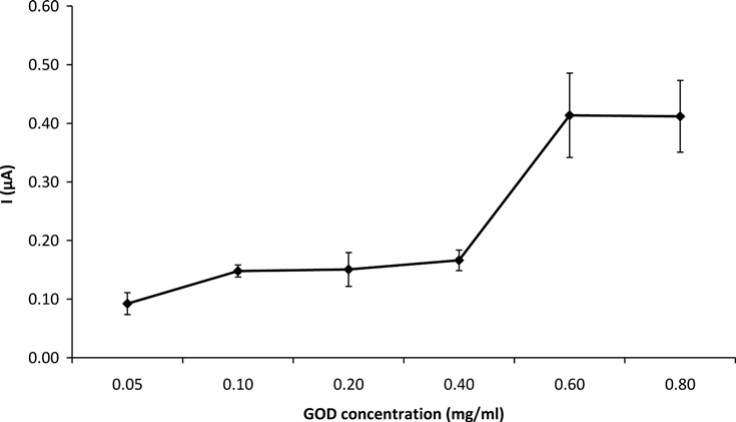
Effect of enzyme concentration used in immobilization on GOD-CHIT/PT response to 4.76 mM glucose. Mean ± SD, n = 6.

**3.2.4 Effect of the enzyme concentration used in immobilization on biosensor response.** The amount of the enzyme used in immobilization was varied from 0.05 to 0.80 mg. The effect of enzyme concentration on the response of the GOD-CHIT/PT to 4.76 mM glucose is shown in Fig. 4. The GOD-CHIT/PT response to glucose was relatively low at enzyme below 0.6 mg GOD concentration. Its response increased by about 60% with 0.4–0.6 mg of glucose, where it showed an optimal response. The response plateaued from 0.6–0.8 mg.

The oxidation of β-D-glucose was rapid at high enzyme concentrations. However, the activity of the immobilized enzyme was decreased by a further increase in the amount of immobilized enzyme due to overloading of the support.

**Fig 5:**
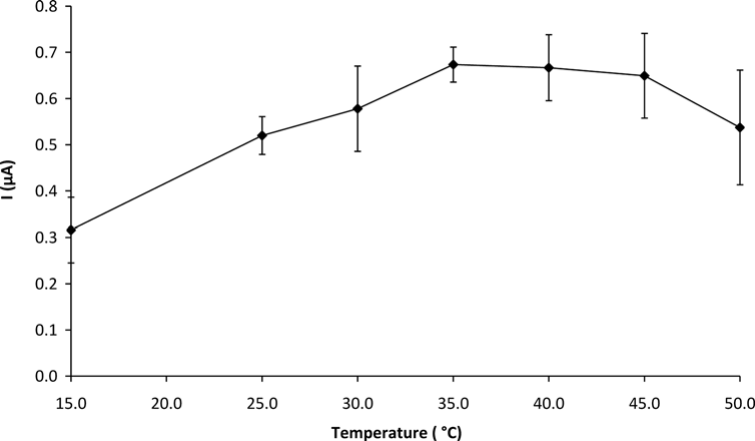
Effect of temperature on GOD-CHIT/PT response to 5.66 mM glucose. Mean ± SD, n = 6.

**3.2.5 Effect of temperature on biosensor response.** The effect of temperature on the response of the GOD-CHIT/PT was investigated from 15 to 50°C. As shown in Fig. 5, the response of the biosensor increased with increasing temperature from 15 to 35°C. There was a slight decrease in the response from 35 to 45°C and a drastic drop in the response was observed from 45 to 50°C. The enzyme might have denatured at the higher temperature and lost its activity. A constant temperature of 35°C was therefore chosen for all subsequent experiments.

**Fig 6:**
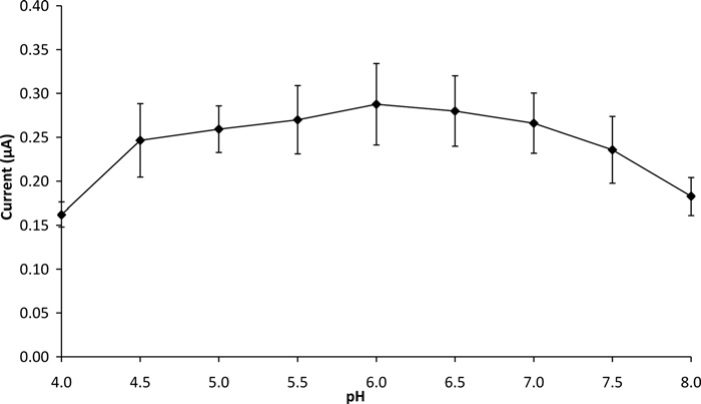
Effect of pH on GOD-CHIT/PT response to 5.66 mM glucose. Experiments were performed at 35°C. Mean ± SD, n = 4.

**3.2.6 Selection of pH for biosensor analysis.** The influence of pH from 4.0–8.0 on the biosensor response to glucose was investigated at 35°C. In general, enzymatic activity is pH-dependent. This enzyme is most stable between pH 3.5 and 8.0 and loses its activity rapidly at pH >8 or pH <2. Fig. 6 shows the anodic current generated by the biosensor at a glucose concentration of 5.66 mM had a maximum current value of 0.29 μA at pH 6.0.

The optimum pH for free GOD is 5.5 [25]. However, in the case of the immobilized enzyme in the biosensor, the optimum pH was shifted to 6.0. Yang et al. [17] explained the pH shift was attributable to the substantial difference in the ionic environment of the matrix around the enzyme’s active sites. Moreover, chitosan membrane becomes cationic at pH values below 6.0 due to the presence of amino groups; thus, chitosan is less stable below pH 6.0 [26]. At pH values higher than 6.0, the catalytic activity of GOD might decrease due to irreversible denaturation of the enzyme [27]. A pH value of 6.0 was thus chosen for subsequent experiments.

**Fig 7:**
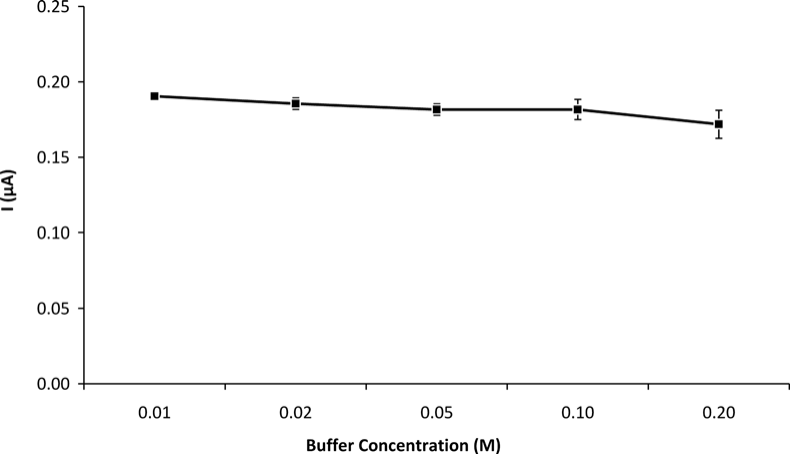
Effect of buffer concentration (pH 6.0) on GOD-CHIT/PT response. The experiments were performed using 5.66 mM glucose at 35°C. Mean ± SD, n = 4.

**3.2.7 Effect of buffer concentration on biosensor response.** The performance of the biosensor in buffer concentrations ranging from 0.01 M to 0.20 M is illustrated in Fig. 7. The maximum response of the enzyme electrode was observed at a buffer concentration of 0.01 M. However, at low buffer concentrations the noise level increased substantially, with the biosensor taking a longer time to reach a steady-state. Since the signal obtained at buffer concentrations from 0.01 M to 0.10 M was not significantly different, 0.10 M phosphate buffer was selected for subsequent studies to obtain the best sensitivity and signal-to-noise ratio.

### 3.3. Calibration of the biosensor

**Fig 8:**
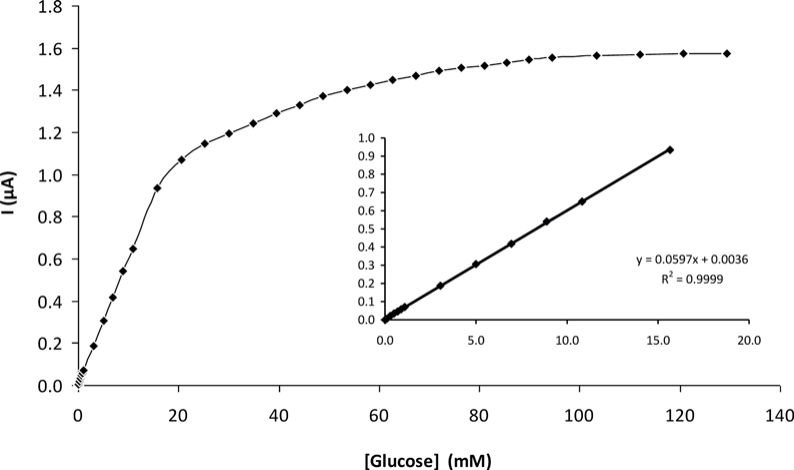
Calibration curve of the GOD-CHIT/PT under optimal experimental conditions. Inset: linear range from 0.01–15 mM glucose.

**Fig 9:**
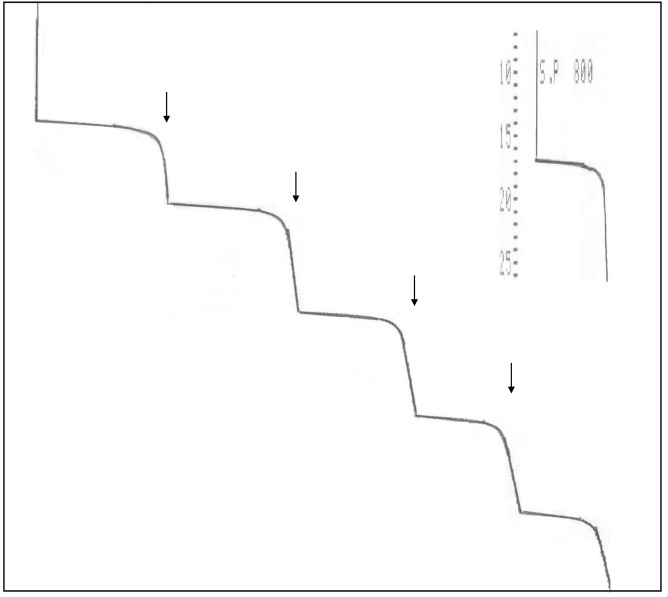
The hydrodynamic response of the GOD-CHIT/PT in a stirred phosphate buffer (0.1 M, pH 6) upon injection of 1.5 mM glucose each step.

A calibration curve for the glucose biosensor was obtained with analyte concentrations from 0.01 to 130 mM under optimal experimental conditions (Fig. 8). With increasing glucose concentrations, more H_2_O_2_ was correspondingly liberated from the enzyme-based reaction, thus resulting in a higher anodic current. The current reached a plateau at a saturating concentration of glucose. The typical current-time plot for the biosensor upon successive stepwise addition of glucose is shown in Fig. 9. The time to reach steady-state current was about 60 s as shown in Fig. 8, inset.

The calibration was linear from 0.01–15 mM glucose, with a slope over the initial linear range of 0.0597 μA/mM and a correlation of determination (R^2^) of 0.9999 (Fig. 9, inset). The detection limit (S/N>3) of 0.05 mM was experimentally obtained with a sensitivity of 50 nA/mM.

**Fig 10:**
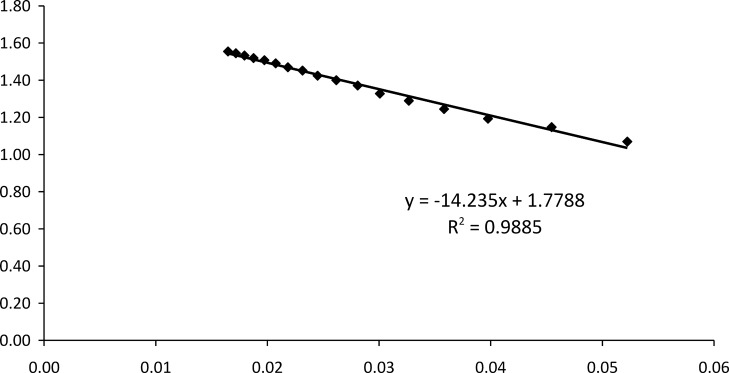
Eadie-Hofstee plot of GOD-CHIT/PT.

The apparent Michealis-Menten constant ($K_{M}^{app}$) is an indication of enzymatic affinity. It can be calculated for immobilized enzymes by the amperometric method because the biosensor response is kinetic [28]. The $K_{M}^{app}$ and *I*_max_ values for the enzyme electrode were found to be 14.2350 mM and 1.7788 μA, respectively, from the Eadie-Hofstee plot (Fig. 10). The $K_{M}^{app}$ value of the biosensor was lower when compared to the value of 23.30 mM reported by Chen et al. [29] and 32.71 mM reported by Xu and Chen [30]. The smaller the $K_{M}^{app}$ value, the stronger the affinity between enzyme and substrate, implying that the present electrode exhibits a higher affinity for glucose.

### 3.4 Repeatability and reproducibility

A reliable glucose biosensor should show good precision (repeatability and reproducibility). Repeatability refers to the agreement between successive measurements of the same sample, whereas reproducibility describes the closeness of agreement between results (signals) obtained using the same method under different conditions (using different glucose biosensors) [31].

Repeated 20 measurements of 3.98 mM glucose by a biosensor had a precision (RSD) of 2.30%, with a mean current of 0.22 μA. The mean value of the current measured by six different biosensors was 0.23 μA and the precision (RSD) was 3.70%.

### 3.5 Storage stability study

**Fig 11:**
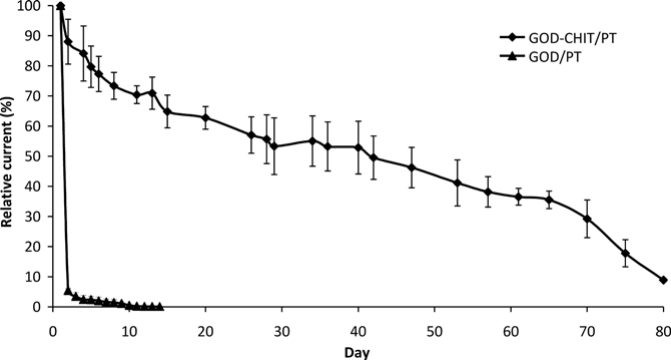
Storage stability of GOD-CHIT/PT biosensors and the comparison of GOD/PT biosensors. Mean ± SD, n = 3.

Enzyme stability within the matrix is a vital consideration in developing a biosensor. As such, the storage stability of the biosensors was evaluated over a period of 80 days (Fig. 11). The GOD-CHIT/PT biosensors retained 55% of their initial activity after being investigated intermittently over a period of 35 days. After 2 months of investigation, the GOD-CHIT/PT biosensors retained about 36% of the initial activity. Beginning on day 65, the activity of the GOD-CHIT/PT biosensors dropped drastically, to less than 10% of the initial activity on day 80. In contrast, the GOD/PT biosensors had lost 95% of their activity on only the second day of the trial.

The results implied that the immobilized enzyme on chitosan membrane could be used repeatedly for amperometric detection over a much longer period compared to free enzyme that was used only once. Crosslinking not only permits high enzyme loading but also improves the stability of the enzyme-chitosan membrane [23]. This is because the aldehyde groups of glutaraldehyde and amine groups on the chitosan or GOD can be easily crosslinked to form Schiff base linkages (-C = N) [32].

However, there was still some loss of activity during the investigation period. We suggest that the gradual decrease in the current might be due to temperature changes in the enzyme electrodes occurring between storage and experimental temperatures (4°C and 35°C, respectively). In addition, partial enzyme denaturation might have occurred over a period of time. The possibility of electrode fouling during storage could also affect the sensitivity of the biosensors.

### 3.5 Glucose determination in fruit

**Fig 12:**
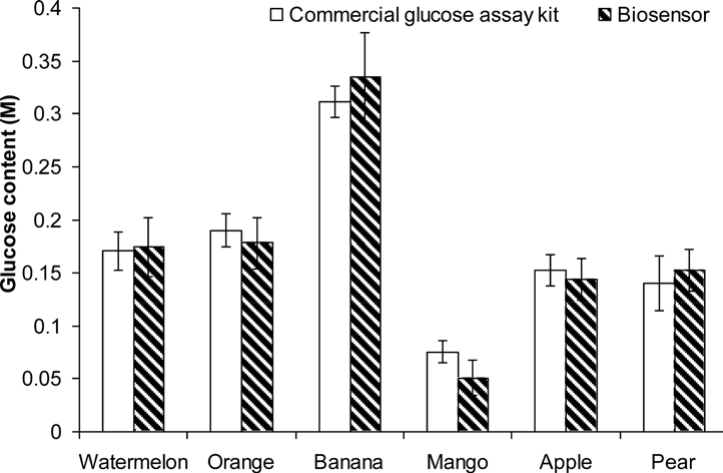
Comparison of commercial glucose assay and biosensor on fruits glucose content measurement. The points shown are the mean of three replicates (± SD).

Both the commercial glucose assay and the biosensor were able to measure the glucose content of fruit homogenates accurately. Statistical analysis showed that there were no significant differences between these two methods of measurement (Fig. 12). Thus, it is suggested the present immobilization method and measurement procedure are reliable and have potential for commercial application.

## Conclusion

Chitosan is a suitable matrix for glucose oxidase immobilization via glutaraldehyde crosslinking. The resulting glucose biosensor shows good repeatability and reproducibility. The results of a storage stability trial suggest that the immobilization process permits the enzyme to be reused, resulting in operational stability over a period of time. The wide linear detection range provided good accuracy to the glucose content measurements. The small value of the Michaelis-Menten constant ($K_{M}^{app}$) implies that the immobilized enzyme has strong affinity for glucose, thus resulting in a more sensitive glucose biosensor in amperometric detection. The introduced glucose biosensor is reliable and economical for agricultural use.

## References

[1] Chen C, Jiang Y, Kan J (2006) A noninterference polypyrrole glucose biosensor. Biosensors and Bioelectronics, 22:639–643. 16540308 10.1016/j.bios.2006.01.023

[2] Thévenot DR, Toth K, Durst RA, Wilson GS (2001) Electrochemical biosensors: recommended definitions and classification. Biosensors and Bioelectronics, 16:121–131. 11261847 10.1016/s0956-5663(01)00115-4

[3] Hu S, Luo J, Cui D (1999) An enzyme-chemically modified carbon paste electrode as a glucose sensor based on glucose oxidase immobilized in a polyaniline film. Analytical Sciences, 15:585–588. 10341421

[4] Gamburzev S, Atanasov P, Wilkins E (1996) Performance of glucose biosensor based on oxygen electrode in physiological fluids and at body temperature. Sensors and Actuators B, 30:179–183.

[5] Mishima Y, Motonaka J, Maruyama K, Nakabayashi I, Ikeda S (2000) Glucose sensor based on titanium dioxide electrode modified with potassium hexacyanoferrate (III). Sensors and Actuators B, 65:343–345.

[6] Ward WK, Jansen LB, Anderson E, Reach G, Klein JC, et al (2002) A new amperometric glucose microsensor: in vitro and short-term in vivo evaluation. Biosensors and Bioelectronics, 17:181–189. 11839471 10.1016/s0956-5663(01)00268-8

[7] Wilson GS, Gifford R (2005) Biosensors for real-time *in vivo* measurements. Biosensors and Bioelectronics, 20:2388–2403. 15854814 10.1016/j.bios.2004.12.003

[8] Low SB, Lee YH, Yamin BM, Ahmad M (2005) Photocurable ferrocene-containing poly(2-hydroxyl ethyl methacrylate) films for mediated amperometric glucose biosensor. Thin Solid Films, 477:104–110.

[9] Xue H, Sun W, He B, Shen Z (2003) Single-wall carbon nanotubes as immobilization material for glucose biosensor. Synthetic Metals, 135–136:831–832.

[10] Lim SH, Wei J, Lin J, Li Q, You JK (2005) A glucose biosensor based on electrodeposition of palladium nanoparticles and glucose oxidase onto Nafion-solubilized carbon nanotube electrode. Biosensors and Bioelectronics, 20: 2341–2346. 15797337 10.1016/j.bios.2004.08.005

[11] Yang Y, Yang H, Yang M, Liu Y, Shen G, et al (2004) Amperometric glucose biosensor based on a surface treated nanoporous ZrO_2_/Chitosan composite film as immobilization matrix. Analytica Chimica Acta, 525:213–220.

[12] Singhal R, Takashima W, Kaneto K, Samanta SB, Annapoorni S, et al (2002) Langmuir-Blodgett films of poly(3-dodecyl thiophene) for application to glucose biosensor. Sensors and Actuators B, 86:42–48.

[13] Krajewska B (2004) Application of chitin- and chitosan-based materials for enzyme immobilizations: a review. Enzyme and Microbial Technology, 35:126–139.

[14] Hsieh BC, Cheng TJ, Wang TY, Chen RL (2003) Use of chitosan membrane from the carapace of the soldier crab *Mictyris brevidactylus* for biosensor construction. Marine Biotechnology, 5:119–125. 12876646 10.1007/s10126-002-0094-x

[15] Wang G, Xu JJ, Ye LH, Zhu JJ, Chen HY (2002) Highly sensitive sensors based on the immobilization of tyrosinase in chitosan. Bioelectrochemistry, 57:33–38. 12049754 10.1016/s1567-5394(01)00174-8

[16] Wang G, Xu JJ, Chen HY, Lu ZH (2003) Amperometric hydrogen peroxide biosensor with sol-gel/chitosan network-like film as immobilization matrix. Biosensors and Bioelectronics, 18:335–343. 12604250 10.1016/s0956-5663(02)00152-5

[17] Yang YM, Wang JW, Tan RX (2004) Immobilization of glucose oxidase on chitosan-SiO_2_ gel. Enzyme and Microbial Technology, 34:126–131.

[18] Yusof NA, Ahmad M (2002) A flow cell optosensor for determination of Co (II) based on immobilized 2-(4-pyridylazo) resorcinol in chitosan membrane by using stopped flow, flow injection analysis. Sensors and Actuators B, 86:127–133.

[19] Zhang M, Li XH, Gong YD, Zhao NM, Zhang XF (2002) Properties and biocompatibility of chitosan films modified by blending with PEG. Biomaterials, 23:2641–2648. 12059013 10.1016/s0142-9612(01)00403-3

[20] Zhou GJ, Wang G, Xu JJ, Chen HY (2002) Reagentless chemiluminescence biosensor for determination of hydrogen peroxide based on the immobilization of horseradish peroxidase on biocompatible chitosan membrane. Sensors and Actuators B, 81:334–339.

[21] Muzzarelli RAA (1983) Chitin and its derivatives: New trends of applied research. Carbohydrate Polymers, 3:53–75.

[22] Lin J, Qu W, Zhang S (2007) Disposable biosensor based on enzyme immobilized on Au-chitosan-modified indium tin oxide electrode with flow injection amperometric analysis. Analytical Biochemistry, 360:288–293. 17134672 10.1016/j.ab.2006.10.030

[23] Wang HS, Pan QX, Wang GX (2005) A biosensor based on immobilization of horseradish peroxidase in chitosan matrix cross-linked with glyoxal for amperometric determination of hydrogen peroxide. Sensors, 5:266–276.

[24] Magalhães JMCS Machado AASC (1998) Urea potentiometric biosensor based on urease immobilized on chitosan membranes. Talanta, 47:183–191. 18967317

[25] Bright H, Appleby M (1969) The pH dependence of the individual steps in the glucose oxidase reaction. The Journal of Biological Chemistry, 244:3625–3634. 5794229

[26] Muzzarelli RAA (1973). Natural chelating polymers. Oxford: Pergamon Press.

[27] Li J, Tan SN, Ge H (1996) Silica sol-gel immobilized amperometric biosensor for hydrogen peroxide. Analytica Chimica Acta, 335:137–145.

[28] Shu FR, Wilson GS (1976) Rotating ring-disk enzyme electrode for surface catalysis studies. Analytical Chemistry, 48:1679–1686. 970629 10.1021/ac50006a014

[29] Chen C, Jiang Y, Kan J (2006) A noninterference polypyrrole glucose biosensor. Biosensors and Bioelectronics, 22: 639–643. 16540308 10.1016/j.bios.2006.01.023

[30] Xu JJ, Chen HY (2000) Amperometric glucose sensor based on glucose oxidase immobilized in electrochemically generated poly(ethacridine). Analytica Chimica Acta, 423:101–106.

[31] Karnes HT, Shiu G, Shah VP (1991) Validation of bioanalytical methods. Pharmaceutical Research, 8:421–426. 1871036 10.1023/a:1015882607690

[32] Sakuragawa A, Taniai T, Okutani T (1998) Fluorometric determination of microamounts of hydrogen peroxide with an immobilized enzyme prepared by coupling horseradish peroxidase to chitosan beads. Analytica Chimica Acta, 374:191–200.

